# The metabolome and bacterial composition of high-moisture Italian ryegrass silage inoculated with lactic acid bacteria during ensiling

**DOI:** 10.1186/s13068-023-02346-8

**Published:** 2023-05-27

**Authors:** Guang-hao Xia, Chang-rong Wu, Ming-zhu Zhang, Feng Yang, Chao Chen, Jun Hao

**Affiliations:** 1 College of Animal Science, Guizhou University, Guiyang, 550025 China; 2 GuiZhou Grassland Technology Extending Station, Guiyang, 550025 China; 3 Key Laboratory of Animal Genetics, Breeding and Reproduction in the Plateau Mountainous Region, Ministry of Education, Guizhou University, Guiyang, 550025 China

**Keywords:** Italian ryegrass, High-moisture silage, Metabolome, Bacterial community, Inoculants

## Abstract

**Background:**

With its high nutritional value and productivity, Italian ryegrass as a biomass feedstock constantly supplies rumen degradable nitrogen and digestible fiber to ruminants. However, biofuel production is easily reduced during ensiling due to the high-moisture content of Italian ryegrass, leading to economic losses. Lactic acid bacteria inoculants could improve lignocellulosic degradation and fermentation quality and decrease dry matter loss during the bioprocessing of silage. Therefore, this study analyzed the effects of *Lactobacillus buchneri* TSy1-3 (HE), *Lactobacillus rhamnosus* BDy3-10 (HO), and the combination of HE and HO (M) on fermentation quality, bacterial community and metabolome in high-moisture Italian ryegrass silage during ensiling.

**Results:**

The results showed that the pH value was significantly lower in the HO groups than in the other treatments at the end of ensiling, and the dry matter and acetic acid contents were significantly higher in the HO group than in the other inoculated groups. All inoculants decreased the diversity of the bacterial community and significantly increased the relative abundance of *Lactobacillus*. Inoculation with HO significantly improved the concentrations of organic acids, dipeptides, ferulic acid, apigenin, and laricitrin. Compared with *Lactobacillus buchneri* TSy1-3 (HE), HO significantly upregulated the flavonoid compounds in the flavone and flavonol biosynthesis pathway.

**Conclusions:**

Overall, these findings suggest that inoculation with HO was beneficial for the development of Italian ryegrass as a biomass feedstock, improving fermentation quality, accelerating changes in bacterial community composition and increasing biofunctional metabolites in high-moisture Italian ryegrass silage.

## Background

Italian ryegrass (*Lolium multiflorum Lam.*) is an important silage crop worldwide and is widely distributed in temperate, tropical and subtropical regions [1]. With its high nutritional value and productivity, Italian ryegrass constantly supplies rumen degradable nitrogen and digestible fiber to ruminants [2]. Although Italian ryegrass has been proven to be suitable for ensiling by previous studies, the nutrient quality is easily reduced during ensiling due to the high-moisture content of Italian ryegrass, leading to economic losses [3, 4]. Traditionally, wilting before ensiling is a desirable way to increase dry matter and to decrease effluent loss during fermentation. However, the intensive silage production area and humid climate in southern China are not well suited for wilting silage crops [4, 5]. In addition, mixing Italian ryegrass with dry crops is another good approach to avoid the negative effect of high-moisture silage [6]. However, the lack of beneficial bacteria in mixed forages is a major limitation of good fermentation [7].

Lactic acid bacteria (LAB) can be used as inoculants to accelerate lactate fermentation, improve animal performance, and regulate bacterial communities [8]. High-moisture alfalfa inoculated with LAB promoted the growth of desirable LAB and reduced the abundance of unfavorable microorganisms such as *Garciella* and *Anaerosporobacter* [9]. However, Torres et al. reported that applying microbial inoculants to high-moisture grain silage had no significant effect on silage quality [10]. The reason might be related to the fact that those silage additives are not specific to the characteristics of high-moisture silage fermentation. LAB selected from various environments play different roles in silage fermentation [11]. Therefore, LAB isolated from high-humidity conditions may better improve the fermentation quality of high-moisture silage. In our previous study, two potentially excellent strains of LAB (*Lactobacillus buchneri* TSy1-3 and *Lactobacillus rhamnosus* BDy3-10) were isolated from a humid climate area in southern China [12]. *Lactobacillus buchneri* is a strain of heterofermentative bacteria, that promotes aerobic stability by fermenting lactic acid to acetic acid and 1,2-propanediol, while *Lactobacillus rhamnosus* is a strain of homofermentative bacteria, that improves the fermentation quality by rapidly producing lactic acid and decreasing the pH [13, 14]. However, it is still unknown whether TSy1-3 and BDy3-10 work very well and how they contribute to the fermentation and bacterial composition of high-moisture Italian ryegrass during ensiling.

Metabolomics techniques, as a sought-after analytical approach, have been widely used to analyze the dynamic changes in metabolites in animals, plants, and fermented food for over a decade [15, 16]. Recently, metabolomics has brought novel insights into silage research. The metabolome and microbiome could be used to investigate the dynamic changes in the bacterial community, metabolites and metabolic pathways during ensiling, and to further explore the underlying fermentation mechanism [17]. According to research on metabolites, bioactive chemicals such as flavonoids, and alkaloids are abundant in paper mulberry leaf silage [18]. Moreover, whole-crop corn silage inoculated with LAB increased the biofunctional metabolites of organic acids, amino acids and phenolic acids [14]. To the best of our knowledge, however, the metabolome in high-moisture Italian ryegrass silage is still unclear.

Therefore, the aim of this study was to investigate the fermentation quality of high-moisture Italian ryegrass silage inoculated with *Lactobacillus buchneri* TSy1-3 and *Lactobacillus rhamnosus* BDy3-10, and to determine the underlying mechanism associated with the contribution of different LAB to the fermentation of high-moisture Italian ryegrass silage by microbiome and metabolome analyses.

## Results

### Characteristics of fresh and ensiled high moisture Italian ryegrass.

The characteristics of fresh Italian ryegrass and silage are shown in Table 1. Overall, there were significant effects of ensiling time and treatment interactions on pH, and lactic acid (LA), acetic acid (AA), propionic acid (PA), and butyric acid (BA) contents (*P* < 0.001). The pH value in the control (CK) group gradually decreased until the end of ensiling, while that in the inoculated groups rapidly decreased during the first 15 days and then increased after 60 days of ensiling. Compared to the other groups, the pH in the HO group was significantly lower at 60 days of fermentation (*P* < 0.05). The dry matter (DM) content in HO was higher than that in the other treatments after 60 days of ensiling, while the lowest DM content was found in HE (*P* < 0.05). The water-soluble carbohydrates (WSC) content of all groups showed a constantly decreasing trend with the process of ensiling (*P* < 0.001). However, the WSC content was not significantly affected by the treatments (*P* = 0.102). The inoculations and ensiling days had no effect on the acid detergent fiber (ADF) and neutral detergent fiber (NDF) contents (*P* > 0.05).

**Table 1:** **C**haracteristics of high-moisture Italian ryegrass before and during ensiling

		pH	LA	AA	PA	BA	DM	WSC	ADF	NDF
			g/kg DM	g/kg DM	g/kg DM	g/kg DM	g/kg	g/kg DM	g/kg DM	g/kg DM
Fresh forage		6.73	–	–	–	–	177.41	176.63	53.89	181.33
Silage										
Day	Treatments									
3	CK	6.66a	11.35Ca	0.39c	0.04Bb	1.25	195.94ab	71.05	94.40	201.83
	HE	6.69a	16.86Ab	0.45b	1.25A	0.06	205.41a	80.62a	96.95	186.26
	HO	6.66a	13.26BCb	0.50b	1.43Ab	ND	204.33a	96.71	105.94	184.65
	M	6.57a	15.24ABb	0.39c	1.49A	ND	198.49	79.06	95.77	186.79
15	CK	6.18Ab	5.49Db	1.20BCb	0.05b	0.38A	205.04Aa	67.65A	90.22	163.00B
	HE	4.12Bc	10.70Bc	1.59Aa	ND	0.15C	179.44Bb	34.42Bb	105.14	189.61B
	HO	3.82Dc	7.98Cc	0.87Cb	0.05c	0.24B	195.37AB	70.05A	128.61	231.07A
	M	3.99Cc	14.47Ab	1.27ABb	ND	0.16C	189.43ABa	46.40B	113.83	205.76AB
60	CK	5.05Ac	6.30Cb	1.77Ba	1.09Ca	ND	189.08Ab	45.31	85.41	163.87
	HE	5.07Ab	21.35Aa	1.79Ba	3.56A	ND	154.09Bc	27.12b	119.64	185.65
	HO	4.53Bb	18.53Ba	2.62Aa	2.48Ba	ND	197.08A	46.05	105.34	188.23
	M	4.93Ab	17.32Ba	2.09Ba	3.29A	ND	165.10Bb	31.29	80.30	162.76
SEM		0.06	0.57	0.102	0.101	0.034	4.931	11.858	12.283	20.778
Levels of significance										
Treatments(T)		***	***	*	***	***	***	0.102	0.118	0.533
Day (D)		***	***	***	***	***	***	***	0.328	0.323
T × D		***	***	***	***	***	**	0.699	0.473	0.537

*LA* lactic acid, *AA* acetic acid, *PA* propionic acid, *BA* butyric acid, *DM* dry matter, *WSC* water-soluble carbohydrates, *ADF* acid detergent fiber, *NDF* neutral detergent fiber, *CK* control, *HE* *Lactobacillus buchneri*, *HO* *Lactobacillus rhamnosus*, *M* combination of HE and HO, *SEM* error of the means, *ND* not detected, *T* treatment, *D* ensiling time, *T × D* interaction of T and D; *P < 0.05; **0.001 < *P* < 0.01; ***P < 0.001. Values with different uppercase letters (A–C) show significant differences among additives in the same ensiling day; values with different lowercase letters (a–c) show significant differences among ensiling days in the same additive (*P* < 0.05).

The concentration of LA in the CK group decreased after 60 days of fermentation, but increased in the inoculated groups. The inoculated groups had significantly higher LA concentrations than the CK group at 60 days of ensiling (*P* < 0.05). The AA content increased in all treatments with prolonged ensiling time, and HO was higher than that in the other treatments at the end of ensiling. The PA concentration of all treatments increased after fermentation for 60 days, while higher PA concentrations were observed in the HE and M groups than in the other groups (*P* < 0.05). BA was not observed in any of the treatments after 60 days of fermentation.

### Bacterial diversity of Italian ryegrass silage during ensiling

**Fig. 1:**
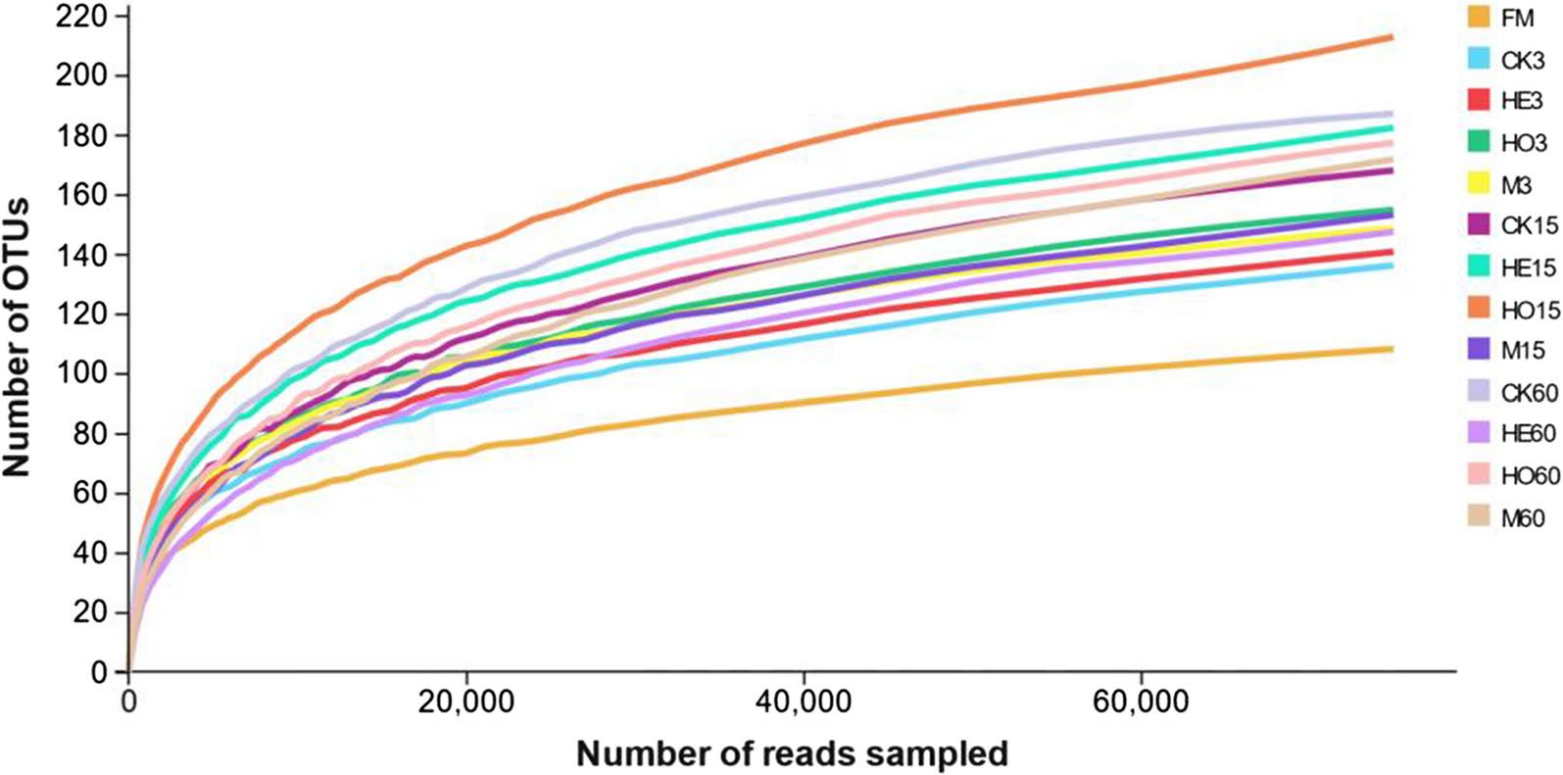
Rarefaction curves for OTUs number in different treatments during high-moisture Italian ryegrass ensiling. *OTUs* number of operational taxonomic units, *FM* fresh material, *CK* control, *HE* *Lactobacillus buchneri*, *HO* *Lactobacillus rhamnosus*, *M* combination of HE and HO; 3, 15, 60: 3, 15, and 60 days of ensiling, respectively

**Fig. 2:**
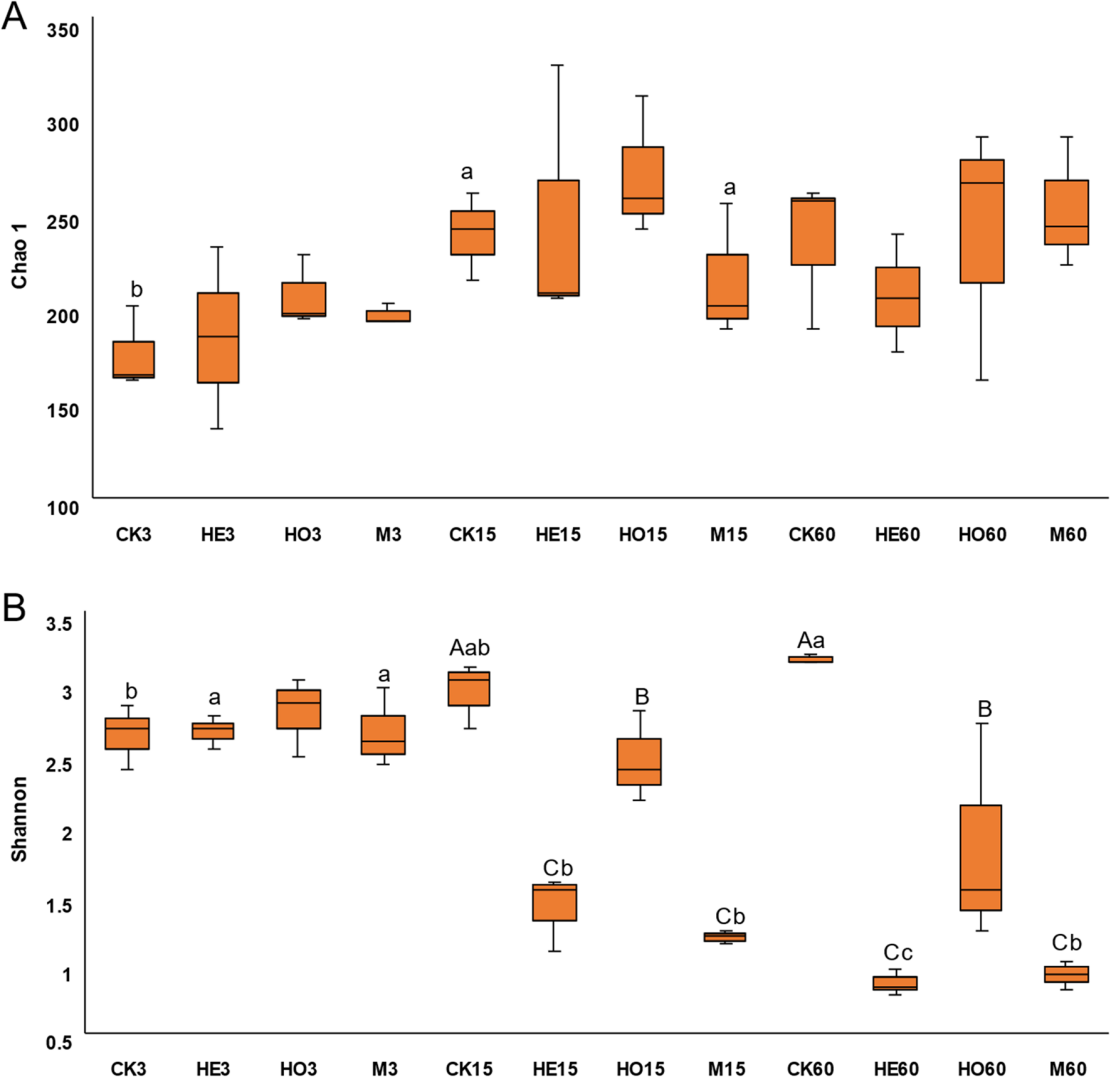
Community diversity and richness of the high moisture Italian ryegrass silages inoculated with or without LAB during ensiling. A Chao l index of silage samples during ensiling; **B** Shannon index of silage samples during ensiling. *CK* control, *HE* *Lactobacillus buchneri*, *HO* *Lactobacillus rhamnosus*, *M* combination of HE and HO; 3, 15, 60: 3, 15, and 60 days of ensiling, respectively. Values with different uppercase letters (A–C) show significant differences among additives in the same ensiling day (*P* < 0.05); values with different lowercase letters (a–c) show significant differences among ensiling days in the same additive (*P* < 0.05)

As shown in Fig. 1, the rarefaction curves of all groups were approach smooth when the sequencing data are great enough with a few new bacterial core operational taxonomic units (OTUs) undetected. The bacterial alpha diversity analysis for fresh forges and each treatment is shown in Fig. 2. The Shannon index in the CK groups tended to rise during ensiling, while those in the HE and M groups tended to decrease. After 60 days of ensiling, lower Shannon index was observed in the HE and M groups, respectively. The Chao 1 index in CK group increased after 15 days of ensiling. However, no significant difference of the Chao 1 index was detected between treatments during ensiling.

**Fig. 3:**
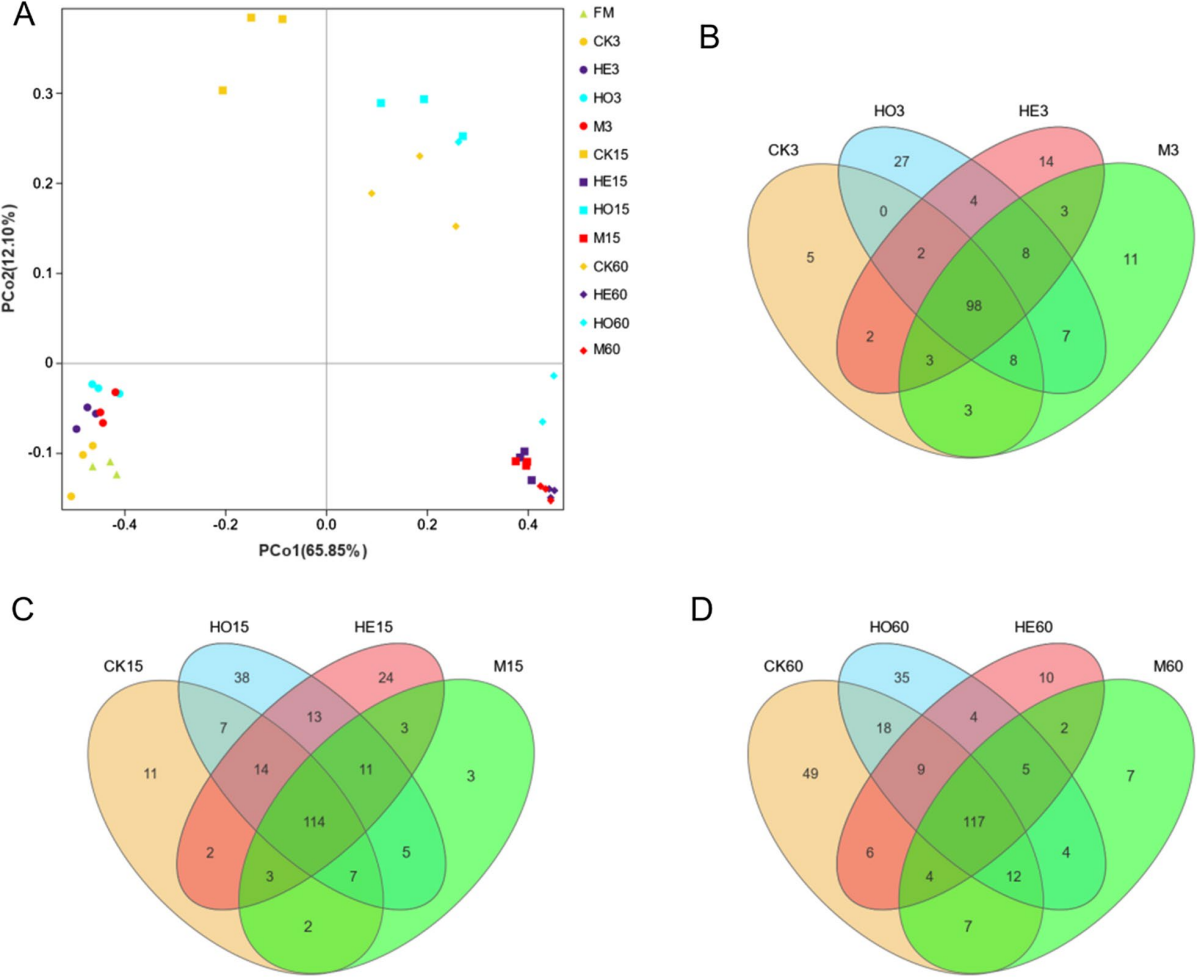
The community dissimilarities of high-moisture Italian ryegrass under different treatments and fermentation time. **A** Principal Coordinate Analysis (PCoA)of the bacterial community of silages in different treatments during ensiling; (**B ~ D**) Venn diagram depicting unique or shared bacterial OTUs in silages under different treatments after 3 (**B**), 15 (**C**), and 60 (**D**) days of ensiling. *FM* fresh material, *CK* control, *HE* *Lactobacillus buchneri*, *HO* *Lactobacillus rhamnosus*, *M* combination of HE and HO; 3, 15, 60: 3, 15, and 60 days of ensiling, respectively

Principal coordinate analysis (PCoA) revealed distinct clusters among groups. As shown in Fig. 3A, PCo1 and PCo2 accounted for 65.85% and 12.10% of the total variance, respectively. The fresh Italian ryegrass and silages ensiled for 3 days were not significantly separated from each other. After 15 days of ensiling, the HO groups were clearly separated, indicating that there were significant differences in microbial communities between the HO and other groups. However, no clear separation and or difference in bacterial communities was found between the HE and M groups. The number of shared OTUs in the Italian ryegrass silage increased during ensiling, and the number of unique OTUs in HO group was more abundant than other groups at the same fermentation time (Fig. 3B–D).

### Bacterial compositions of Italian ryegrass silage during ensiling

**Fig. 4:**
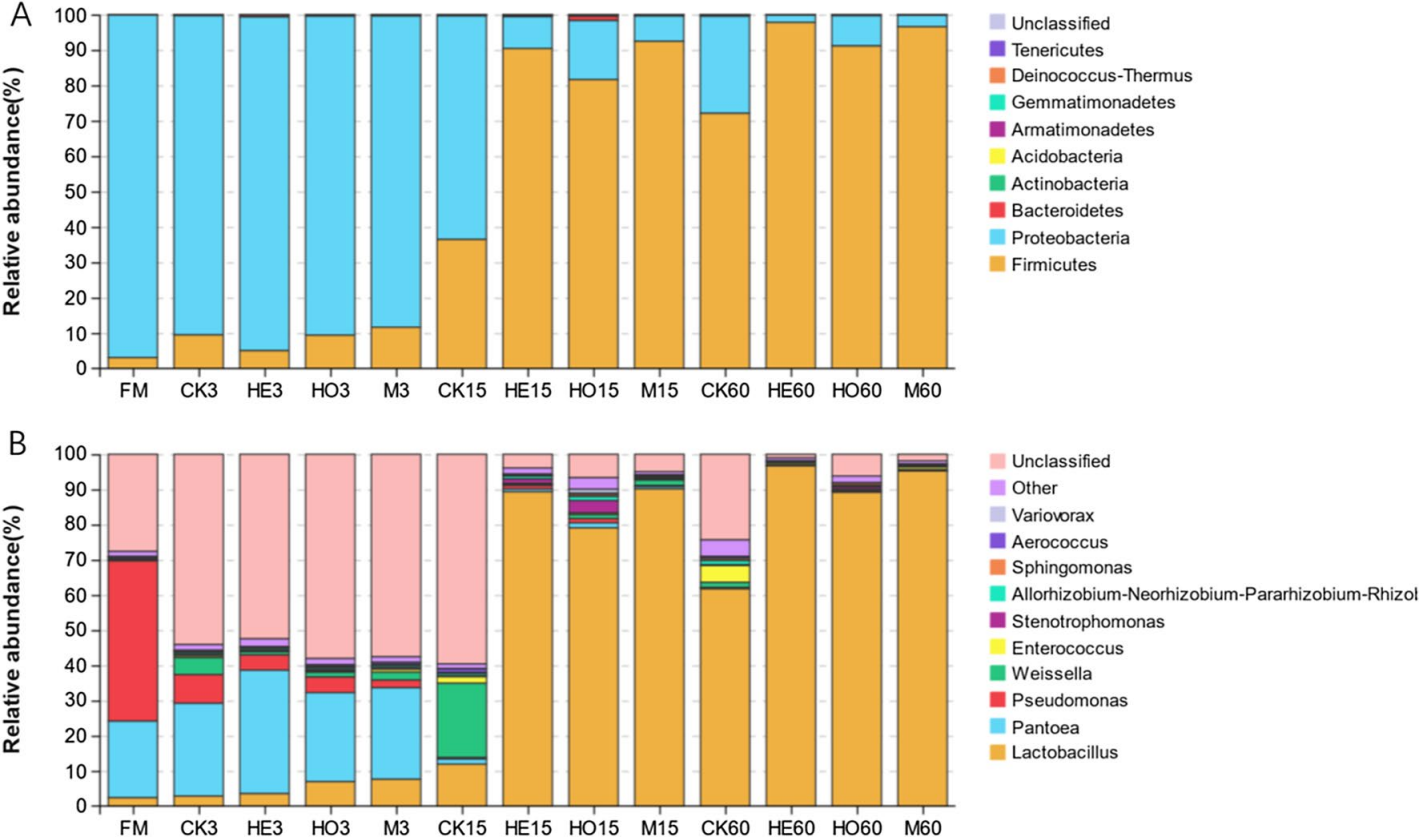
Bacterial composition of high-moisture Italian ryegrass inoculated with or without LAB during ensiling. **A** Relative abundance of bacterial community at phylum level; **B** relative abundance of bacterial community at genus level. *FM* fresh material, *CK* control, *HE* *Lactobacillus buchneri*, *HO* *Lactobacillus rhamnosus*, *M* combination of HE and HO; 3, 15, 60: 3, 15, and 60 days of ensiling, respectively

At the phylum level, Proteobacteria and Firmicutes were mainly detected in the bacterial communities during the ensiling process (Fig. 4A). Proteobacteria dominated the microbial composition in all treatments during the first 3 days of ensiling. However, the abundance of Firmicutes dramatically increased after 15 days of fermentation, and Firmicutes became the dominant bacteria in the LAB-inoculated groups. After 60 days of ensiling, the abundance of Firmicutes in LAB-inoculated groups was significantly higher than that in CK, and the abundance of other microbes at the phylum level in the HO, HE and M groups was less than 10%.

To further investigate the effects of LAB inoculants on the microbial community during fermentation, the bacterial composition of Italian ryegrass silage at the genus level was detected. As shown in Fig. 4B, the epiphytic bacteria in fresh Italian ryegrass mainly included *Pseudomonas*, *Pantoea*, *Lactobacillus, Weissella, Allorhizobium-Neorhizobium-Pararhizobium-Rhizobium* and *Stenotrophomonas*. The *Lactobacillus* abundance increased significantly and became the most abundant bacteria in all groups at the end of ensiling. *Lactobacillus* dominated the bacterial community of the inoculated groups after 15 days of ensiling. In the CK groups, however, it took 60 days for *Lactobacillus* to dominate the bacterial community. Inoculated with LAB significantly increased the relative abundance of *Lactobacillus* when compared to the CK groups, while the difference between the three inoculated groups was not obvious. The relative abundance of *Weissella* in CK increased after 15 days of ensiling and then decreased at the end of fermentation. However, the relative abundance of *Weissella* in the LAB-treated group not only decreased with prolonged ensiling time, but was also lower than that in the CK group at the same ensiling time. The relative abundance of *Pseudomonas* and *Pantoea* notably declined with the prolonged ensiling process. After 60 days of ensiling, *Pantoea* was more abundant than in the inoculated groups. The above results indicate that HO, HE and M altered the bacterial community of Italian ryegrass silage during ensiling.

### Metabolites of Italian ryegrass silage during ensiling

**Fig. 5:**
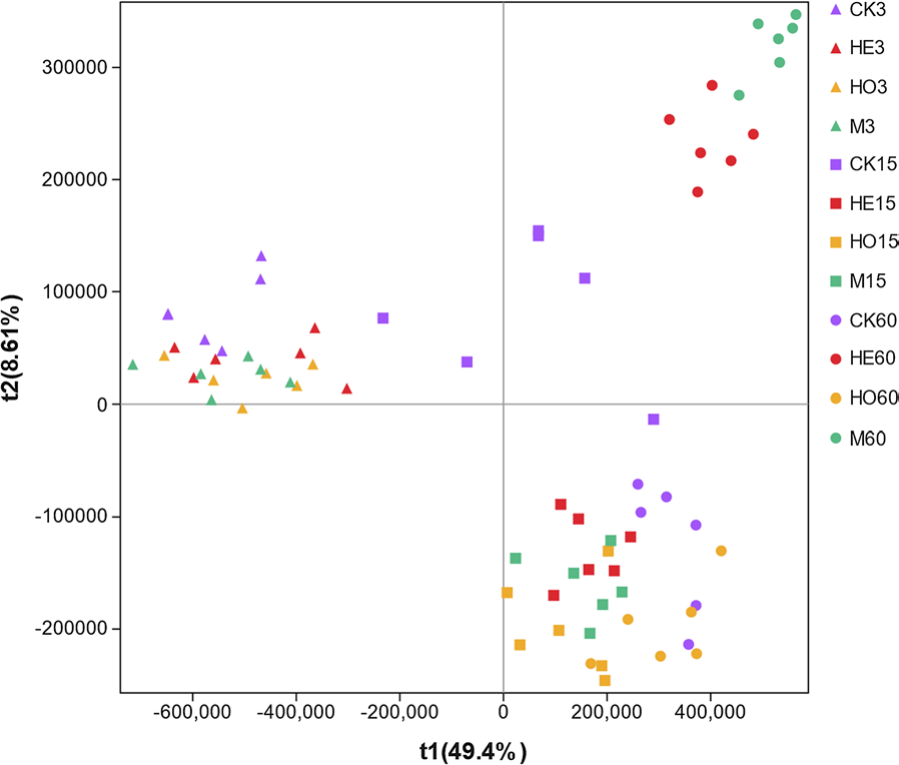
Partial least squares-discriminate analysis of metabolic profiles in Italian ryegrass silage. *CK* control, *HE* *Lactobacillus buchneri*, *HO* *Lactobacillus rhamnosus*, *M* combination of HE and HO; 3, 15, 60: 3, 15, and 60 days of ensiling, respectively

To investigate the dynamic changes in the metabolites of Italian ryegrass silage during ensiling, an untargeted metabolomic approach was used in this study. In total, 2999 metabolites were detected in 72 samples, and 1023 metabolites were annotated. The differences in metabolites among samples were discriminated through a supervised pattern recognition approach to maximize sample separation (Fig. 5). The partial least squares-discriminate analysis score plots explained 58.01% of the total variation between all groups. No clear separation was found in the different groups in the first 3 days of ensiling. However, after 60 days of fermentation, the treatments were significantly separated from each other.

**Fig. 6:**
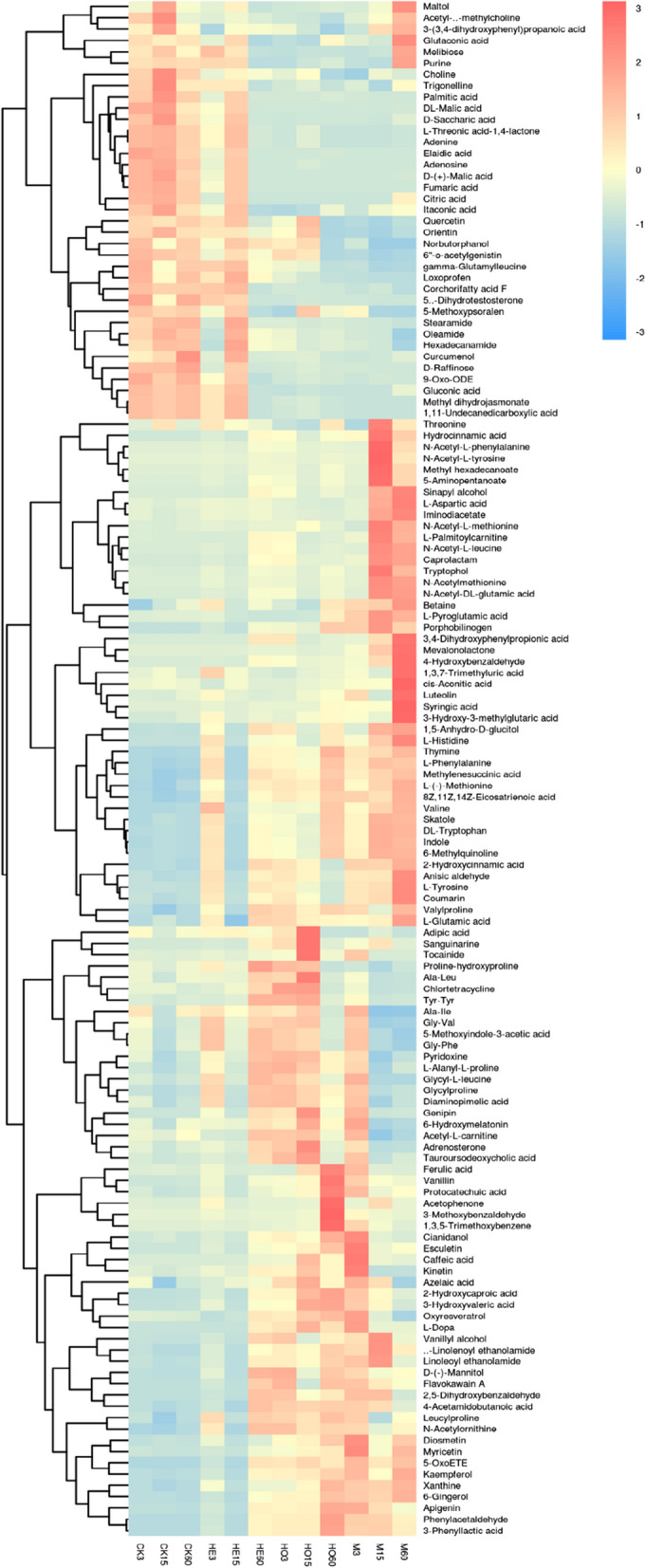
Heatmap of the significantly different metabolites during the ensiling process of Italian ryegrass. *CK* control, *HE* *Lactobacillus buchneri*, *HO* *Lactobacillus rhamnosus*, *M* combination of HE and HO; 3, 15, 60: 3, 15, and 60 days of ensiling, respectively

There were 136 differential metabolites based on the variable importance in projection (VIP > 1), and P values (*P* < 0.05) were detected in this study (Fig. 6), including lipids, carboxylic acids and derivatives, phenols, isoflavonoids, indoles and derivatives, flavonoids, and other metabolites. Inoculation with HO significantly improved the concentration of organic acids such as 2-hydroxycaproic acid, protocatechuic acid, 3-phenyllactic acid and 3-hydroxyvaleric acid. The relative abundance of essential amino acids, such as threonine, methionine, valine and phenylalanine, was rich in the M groups. Dipeptides (Ala-Leu, Tyr-Tyr, Ala-Ile, Gly-Phe, Gly-Val) were abundant in the HO groups during 3–15 days of ensiling. Some metabolites with biological functions were also detected in high-moisture Italian ryegrass silages during fermentation. Inoculation with HO significantly increased the concentrations of ferulic acid, apigenin and laricitrin. Inoculation with M increased the contents of kaempferol, luteolin, syringic acid, caffeic acid and coumarin.

### Metabolic pathways of differential metabolites during ensiling

**Fig. 7:**
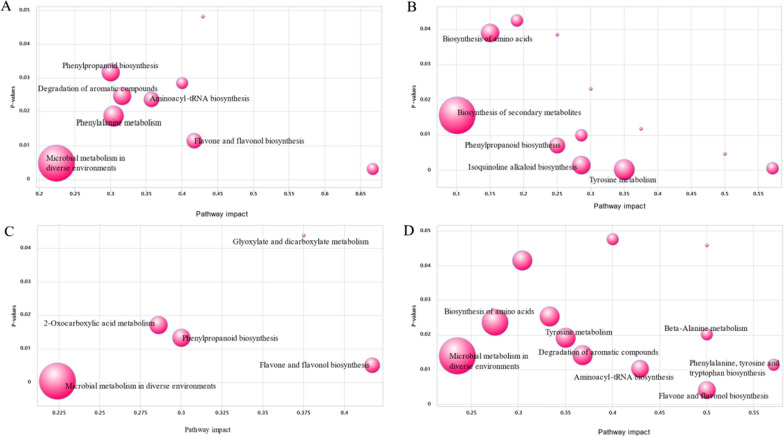
KEGG pathway enrichment analysis of differentially accumulated metabolites. **A** pathway enrichment in CK vs HE; **B** pathway enrichment in CK vs HO; **C** pathway enrichment in CK vs M; **D** pathway enrichment in HE vs HO. The x-axis represents the enrichment factor, while the y-axis represents the *P*-values. The representative circles (varying from small to large) refer to increased numbers of metabolites annotated to pathways

To understand the possible metabolic pathways of differential metabolites, the identified metabolites were annotated in the KEGG database. Through mapping the differential metabolites among CK versus HE, the main affected metabolic pathways (over 5 differential metabolites were annotated to the pathways) were “microbial metabolism in diverse environments”, “phenylalanine metabolism”, “degradation of aromatic compounds”, and “phenylpropanoid biosynthesis” (*P* < 0.05) (Fig. 7A). Compared with CK, the main metabolic pathways (over 5 differential metabolites were annotated to the pathways) involved in HO were “biosynthesis of secondary metabolites”, “isoquinoline alkaloid biosynthesis”, “biosynthesis of amino acids”, and “tyrosine metabolism” (*P* < 0.05) (Fig. 7B). “Microbial metabolism in diverse environments”, “2-oxocarboxylic acid metabolism”, “phenylpropanoid biosynthesis”, “glyoxylate and dicarboxylate metabolism” and “flavone and flavonol biosynthesis” were the significantly affected metabolic pathways in the M treatment when compared with CK (*P* < 0.05) (Fig. 7C). Based on statistical significance, the most affected pathways in the HO compared with HE were “flavone and flavonol biosynthesis”, “aminoacyl-tRNA biosynthesis”, “phenylalanine, tyrosine and tryptophan biosynthesis”, “microbial metabolism in diverse environments”, and “degradation of aromatic compounds” (*P* < 0.05) (Fig. 7D).

**Fig. 8:**
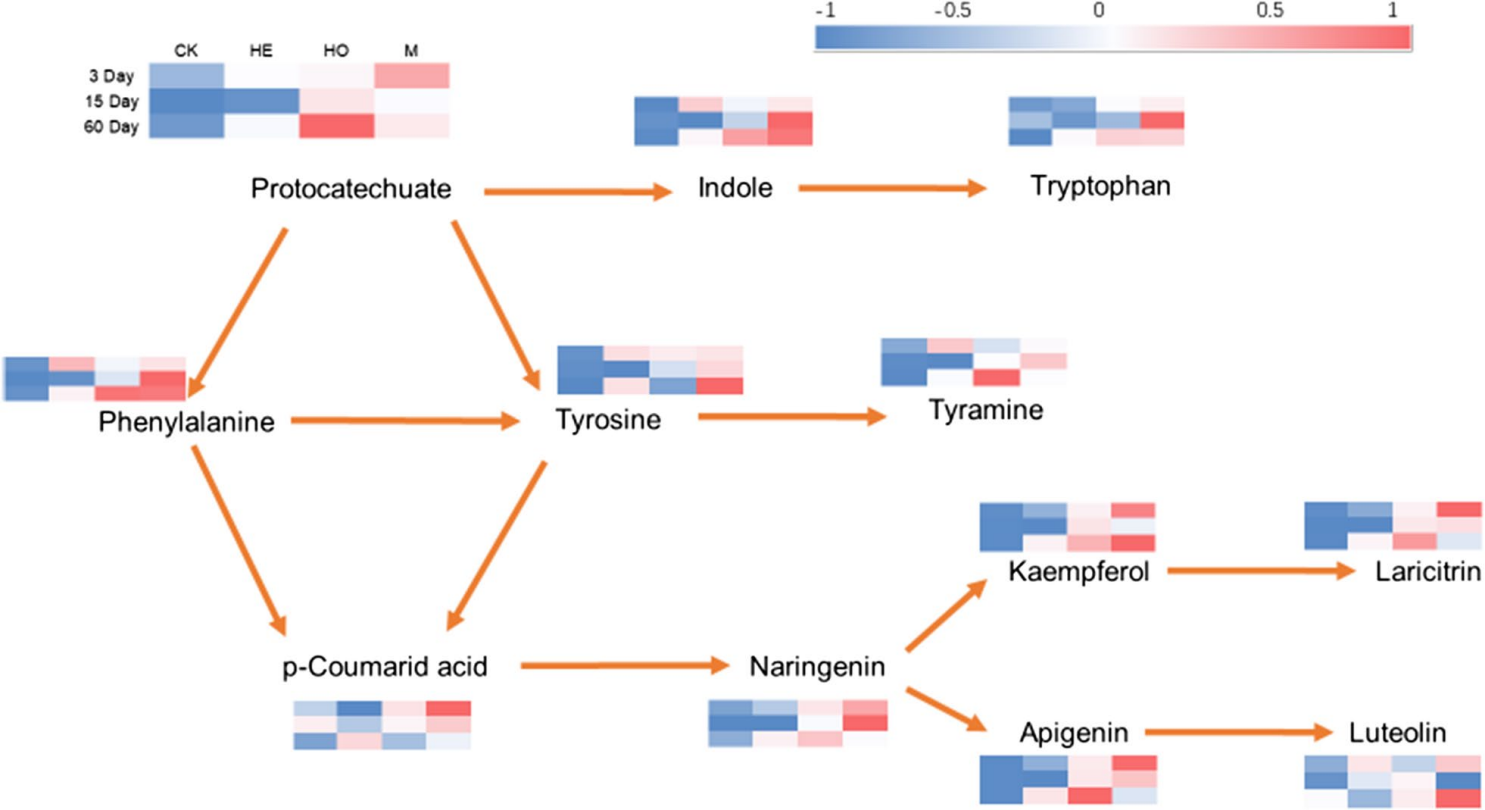
Metabolic pathways associated with differential metabolites variations in the ensiling process of Italian ryegrass silage. *CK* control, *HE* *Lactobacillus buchneri*, *HO* *Lactobacillus rhamnosus*, *M* combination of HE and HO

Amino acid metabolism and biosynthesis of other secondary metabolites based on the KEGG database at level 2 were investigated to uncover metabolite changes in response to prolonged fermentation days and LAB additives (Fig. 8). Tyrosine and phenylalanine are the products of the phenylalanine, tyrosine and tryptophan biosynthesis pathways, and can then be converted into tyramine through tyrosine metabolism and phenylalanine metabolism pathways. p-Coumaric acid was produced by the phenylpropanoid biosynthesis pathway and further generated naringenin. After 60 days of ensiling, the contents of the downstream metabolites produced by the flavone and flavonol biosynthesis pathways, such as laricitrin and luteolin, were rich in the HO and M groups, respectively.

## Discussion

### Effects of LAB inoculants on the fermentation quality of high-moisture Italian ryegrass silage

Normally, high moisture in forage presents difficulty in achieving acceptable fermentation quality [19]. This was proven by the poor fermentation characteristics found in the CK groups, as demonstrated by the high pH value and low LA content during ensiling. The high pH value in the CK groups indicated that the production of ammonia nitrogen and other alkaline substances caused by proteolysis was abundant during ensiling [20]. Interestingly, in the present study, the pH value of the inoculated groups decreased significantly after 15 days of ensiling and then increased at the end of ensiling. This finding indicates that although inoculation with LAB was effective in inhibiting protein degradation, the alkaline substances caused by proteolysis accumulated after 60 days of fermentation. Hence, the above results also illustrated that high-moisture silage may not be conducive to long-term storage [21]. Well fermentation characteristics usually depend on good nutrient preservation in silage. A previous study reported that heterofermentation had a lower DM content than homofermentation [20]. Similarly, in the present study, the fermentation of HE also resulted in more dry matter loss after 60 days of ensiling. This may be because the higher pH environment in HE made yeasts and clostridia still exist, and HE had to consume lots of nutrients to compete with these undesirable bacteria. However, inoculation with HO significantly improved the fermentation quality in this study. The main reason for this result was that homo-LAB played a crucial role in quickly reducing the pH value and further led to reduced forage mass loss. In fact, wilted ryegrass silage inoculated with LAB had higher LA and AA concentrations in a previous study. Li and Nishino reported that after 56 days of ensiling, wilted ryegrass inoculated with homofermentative and heterofermentative LAB showed an LA content of 110 g/kg DM and an AA content of 43.3 g/kg DM [22]. This result may indicate that high-moisture ryegrass silage inhibited the production of organic acids by LAB. Excessive concentrations of PA may result in low fermentation efficiency or secondary fermentation [23]. According to Agarussi et al., the PA content for well-ensiled forage should range from 1 to 10 g/kg DM [24]. After 60 days of fermentation, the PA content in all treatments ranged from 1.09 to 3.56 g/kg DM in this study, which was an acceptable range for high moisture fermentation. BA is a major product produced by clostridial fermentation, and it is considered undesirable because it not only results in nutritional loss but also decreases feed intake by ruminants [18, 25]. However, BA was not detected in any of the treatments at the end of ensiling, indicating that the metabolic activity of clostridia was limited after fermentation.

### Effects of LAB inoculants on the bacterial microbiota in high-moisture ryegrass silages

The quality of silage mainly depends on the composition of the microflora, and the bacterial community varies with the ensiling process [26, 27]. LAB compete with aerobic bacteria, facultative anaerobic bacteria and other undesirable microorganisms during ensiling, resulting in a decrease in bacterial diversity with prolonged anaerobic fermentation [23]. The present study provided further evidence that the Shannon index of the bacterial community decreased in HE and M groups during ensiling. The lower alpha diversity of the bacterial community in the LAB-treated groups indicated that inoculants helped to competitively exclude miscellaneous bacteria. There was no clear separation between the HE and M groups according to the principal coordinates analysis. This result may be due to *Lactobacillus buchneri* maintaining a competitive advantage in the M-treated Italian ryegrass silage.

The relative abundance of Proteobacteria decreased significantly during ensiling, while Firmicutes became the most abundant bacterial phylum. This shift in the bacterial community from Proteobacteria to Firmicutes during ensiling has been reported by previous studies on alfalfa and sugarcane top silage [27–29]. As expected, a significantly lower relative abundance of *Weissella* and *Enterococcus* and a higher relative abundance of *Lactobacillus* were observed in the LAB-treated groups after 15 days of ensiling. Early epiphytic LAB, such as *Weissella*, *Enterococcus,* and *Pediococcus*, are usually rich in the early ensiling stage, then became less vigorous and are replaced by more acidic *Lactobacillus* with prolonged fermentation time [30, 31]. Therefore, HE, HO and M significantly accelerated the process of the bacterial community composition shift in high-moisture Italian ryegrass silage. *Pantoea spp.* belonging to the family Enterobacteriaceae, are well-known agents of disease in forage, and lead to community-acquired urinary tract, skin, soft tissue, and other infections [32]. *Pseudomonas*, a member of the Pseudomonadaceae family, are obligate aerobic and frequently result in mastitis in dairy cattle [33]. The relative abundance of *Pantoea*, and *Pseudomonas* decreased dramatically, and *Lactobacillus* became the most abundant bacteria after 60 days of ensiling. This may be because oxygen was depleted and anaerobic metabolism contributed to a low pH value environment during anaerobic fermentation, which was beneficial to the proliferation of *Lactobacillus* [14, 34]. Furthermore, *Lactobacillus* demonstrated complicated antimicrobial mechanisms on ensilage, including competitive exclusion and the ability to generate antimicrobial compounds, peptides, and bacteriocin [23].

In the present study, the underlying mechanisms by which HO, HE and M alter bacterial communities in high-moisture Italian ryegrass silage may differ. *Lactobacillus rhamnosus* reduced miscellaneous bacteria by rapidly decreasing the pH value, and aciduric *Lactobacillus* dominated the bacterial community of the HO group. However, the undesirable bacteria in the HE and M groups may be directly limited by the antibacterial characteristics of *Lactobacillus buchneri* [23]. Therefore, *Lactobacillus* could dominate the bacterial community of HE and M.

### Effects of inoculants on metabolomic profiles in high-moisture Italian ryegrass silages

A total of 2999 metabolites were detected in high-moisture Italian ryegrass silages, which far exceeded the number of metabolites reported in previous studies of normal moisture silage. Less than 1000 metabolites were found in the whole crop corn silage and paper mulberry leaf silage, and only 214 metabolites were identified from stylo silage [18, 35, 36]. The various types and compositions of metabolites may be due to the different silage material species [14]. However, high moisture ensiling may delay the reduction in pH value, which caused the metabolism of microorganisms to be more active than previous studies. Thus, numerous metabolites were produced in this study.

Metabolome profiles of the current Italian ryegrass silage illustrated that HE, HO and M differentially modulated the metabolite composition during fermentation. Metabolites with beneficial effects could be regarded as an important index to evaluate fermentation properties [35]. Organic acids usually have sourness, special flavor, taste or aroma, and they can improve the palatability of silage [37, 38]. Consistent with Hu et al. (2020), they found that organic acids were more abundant in home-LAB-treated silage than in untreated silage using metabolomics techniques. However, some organic acids, such as palmitic acid, elaidic acid, citric acid, and gluconic acid, in the CK groups were also more abundant than those in the LAB-treated groups during ensiling. This may be caused by the complex conditions in the untreated silage, such as higher microbial diversity and stronger fungal activity [39]. Amino acids are vital nutrients and flavor compounds for both ruminants and humans. The outcomes of amino acids and peptides are mainly due to proteases produced by microbes that degrade the protein during ensiling [40]. In the present study, M dramatically increased the essential amino acids, and HO increased dipeptide concentrations. The production of essential amino acids and dipeptides plays important roles in enhancing the quality of fermentation [41]. Furthermore, this finding may also illustrate that *Lactobacillus rhamnosus* limited the enzymatic activity of microorganisms and inhibited bacteria from consuming the peptides and essential amino acids. These results suggested that inoculation with HO was beneficial for increasing the palatability and nutritional value of high-moisture silage.

Many phenolic compounds, such as phenolic acids, flavonoids and coumarins, were found to be significantly higher in HO and M than in the CK after 60 days of ensiling. The increase in phenolic acids such as ferulic acid, syringic acid and caffeic acid in the LAB-treated group was similar to that reported by He et al., who explained that the increase in phenolic compounds might be because LAB promoted the depolymerization of complex compounds into more bioavailable simple compounds [18]. As a main class of plant secondary metabolites, flavonoids are well known to have beneficial effects on animal health and welfare due to their antioxidative, antidiabetic, antimicrobial, antimutagenic and anticancer properties with low toxicity effects [42, 43]. Moreover, coumarins have been increasingly biomedical applied in recent years for exhibiting antiviral, antitumor, antithrombotic, anti-inflammatory, and vasodilatory activities [44, 45]. Therefore, the above metabolome investigation suggested that inoculation with HO or M is an effective way to improve the multifunctional profiles of ensiled high-moisture forage.

After high-moisture Italian ryegrass was inoculated with LAB, metabolites were subjected to complicated reactions and regulation of molecular pathways that worked in parallel to improve silage quality and biofunctions. Hence, metabolic pathways play an essential role in demonstrating the key metabolite activities and dynamics of metabolism during fermentation [46]. The present study showed that amino acid metabolism, including phenylalanine metabolism, tyrosine metabolism, and biosynthesis of amino acids, was significantly affected by LAB inoculants during fermentation. Tyrosine is regarded as a necessary dietary amino acid for ruminants and humans, and a diet supplemented with phenylalanine is a regular way to compensate for tyrosine [47]. After a series of amino acid metabolism, some of these metabolites were transferred to the biosynthesis of other secondary metabolites. The flavone and flavonol biosynthesis pathway is a fraction of the biosynthesis of other secondary metabolites and was one of the most affected pathways by LAB inoculants in the current study. The flavonoid metabolic pathway belongs to the phenylpropanoid metabolic pathway, which generates massive amounts of polyphenols such as phenolic acids, lignans, stilbenes and lignins [48]. In this study, all differential flavonoid compounds in the flavone and flavonol biosynthesis pathway were upregulated by HO compared with HE, and relatively higher concentrations of kaempferol and luteolin were detected in the M groups after 60 days of ensiling. The good properties of the flavonoid compounds mentioned above helped to limit the proliferation of undesirable microorganisms in silage. Therefore, *Lactobacillus rhamnosus* improved the fermentation quality of high-moisture silage by upregulating flavonoid compounds in the pathway of flavone and flavonol biosynthesis in this study.

## Conclusion

The addition of HO was beneficial for preserving green biomasses and improving fermentation quality during the bioprocessing of high-moisture Italian ryegrass silage. HO, HE and M increased the relative abundance of *Lactobacillus* and decreased the relative abundances of *Pantoea* and *Weissella*. A total of 2999 metabolites were detected in high-moisture Italian ryegrass during ensiling. Inoculation with HO was an effective way to improve the concentrations of organic acids, dipeptides, ferulic acid, apigenin, and laricitrin. HO upregulates flavonoid compounds in the pathway of flavone and flavonol biosynthesis during ensiling. Therefore, homo-LAB is suggested to be inoculated in high-moisture silage to preserve the biomass, improve fermentation quality, and enrich biofunctional metabolites.

## Materials and methods

### Silage preparation

Italian ryegrass was cultivated on an experimental farm in Dushan city (subtropical humid monsoon climate, 25°33′ N, 107°37′ E; Guizhou Province, China) on Oct. 20, 2020. The second growth of the Italian ryegrass row was harvested on January 5, 2021, at the jointing stage of regrowth. The plant material was chopped into 1–2 cm pieces by a precision chopper and immediately transported to the laboratory for further processing. A selected heterofermentative strain HE (*Lactobacillus buchneri* TSy1-3) and a selected homofermentative strain HO (*Lactobacillus rhamnosus* BDy3-10) isolated from karst areas in Guizhou with warm and humid climate were used as additives for silage preparation. The Italian ryegrass was thoroughly mixed and treated as follows: (1) 10 ml/kg distilled water (CK), (2) 10 ml/kg *Lactobacillus buchneri* TSy1-3 (HE), (3) 10 ml/kg *Lactobacillus rhamnosus* BDy3-10 (HO), and (4) 5 ml/kg HE combined with 5 ml/kg HO (M). The application rate of each LAB inoculant was 1 × 10^8^ cfu/g fresh Italian ryegrass. In each polyethylene plastic bag (225 mm × 350 mm, Reelanx Company, Shenzhen, China), 300 g of Italian ryegrass was packed and vacuum sealed. The silos were collected and sampled at 3, 15, and 60 days of fermentation. In total, 36 bags (4 treatments × 3 time points × 3 repeats) were stored at ambient temperature (5–15 °C) in the dark.

### Fermentation analysis

Samples of approximately 100 g from each bag were dried at 65 °C for 48 h in a forced-air oven to determine the DM content. Then, the dried samples were ground to pass a 1 mm screen with a laboratory mill (Hainai ship Hi-100 C, Hainai Yinjiang Litongtrade company Lit., Zhejiang, China). The ground dry samples were used for detecting the contents of WSC. The WSC contents were analyzed by the colorimetric method after reaction with anthrone reagent [49]. The concentrations of NDF and ADF were measured by the methods Hao et al. [50]. 20 g silage was mixed with 180 mL distilled water and stored at 4 °C refrigerator for 24 h and then filtered through four layers of cheesecloth. The pH value was measured via a glass electrode pH meter (Starter 300; Ohaus Instruments Co. Ltd., Shanghai, China). The concentrations of organic acids including LA, AA, PA and BA were determined using high-performance liquid chromatography as described by Cai et al. [51].

### Bacterial community analysis

The fresh Italian ryegrass and silage ensiled for 3, 15, and 60 days were selected for bacterial community analyses. The solution for DNA extraction was centrifuged at 12,000 g for 10 min at 4 °C to form a pellet for subsequent DNA extraction. Microbial DNA was isolated from silage samples using a DNA Isolation Kit (Omega Bio-tek, Norcross, GA, U.S.) according to the manufacturer’s specification. The quality and quantity of extracted DNA were evaluated by the HiPure Soil Kit (QIAGEN, Inc., Venlo, The Netherlands). The V5–V7 regions of the bacterial 16 S rRNA gene were amplified using the specific primers 799F (AACMGGATTAGATACCCKG) and 1193R (CGTCATCCCCACCTTCC). The PCR program was as follows: 3 min of denaturation at 95 °C, 35 cycles of 95 °C for 60 s, 30 s for annealing at 55 °C, 60 s for elongation at 72 °C, with a final extension of 72 °C for 10 min. After PCR products were purified and quantified, the equimolar and paired-end sequencing (PE250) was performed on the Illumina Novaseq 6000 platform (Personal Biotechnology Co., Ltd., Shanghai, China).

All the raw reads were checked using FLASH (version 1.2.11), and low-quality sequences (quality scores below 20) were excluded based on the QIIME quality control process (version 1.7.0). A 97% similarity cutoff was used to define operational taxonomic units (OTUs) using the UPARSE pipeline. The taxonomy assignment of representative sequences was conducted using the Ribosome Database Project classifier (Version 2.2).

### Metabolite analysis

Silage samples were freeze-dried and then ground with a mixer mill. Then, 1 g of powder was transferred to a 10 mL EP tube and extracted with a 500 μL extraction (80% aqueous methanol). The samples were vortexed for 30 s and incubated on ice for 5 min, followed by overnight shaking at 4 ℃. Then, all samples were centrifuged at 15,000*g* for 20 min at 4 °C. A part of the supernatant was diluted with LC–MS grade water to a final concentration containing 53% methanol. The samples were then transferred to fresh EP tubes with 0.22 μm filters, and then centrifuged at 15,000 rpm and 4 °C for 10 min. The resulting supernatants were subjected to Liquid Chromatograph Mass Spectrometer (LC–MS) analysis. In order to evaluate the stability of the analytical system during the investigation, a quality control sample was prepared for every six samples.

The analytical conditions were as follows: HPLC: column, Waters ACQUITY UPLC HSS T3 C18 (1.8 µm, 2.1 mm × 100 mm); water (0.1% formic acid, A) and acetonitrile (0.1% formic acid, B) were used as mobile phase. Sample measurements were performed with a gradient program that employed the starting conditions of 95% A and 5% B. Within 10 min, a linear gradient of 5% A and 95% B was programmed, and a composition of 5% A and 95% B was maintained for 1 min. Subsequently, a composition of 95% A and 5.0% B was adjusted within 0.1 min and maintained for 2.9 min. The speed of the mobile phase was 0.2 mL/min. The column oven temperature was 40 °C, and the injection volume was 4 μL. The qualitative and quantitative analysis of metabolites, and raw data pre-processing were performed according to the methodology described by Wu et al. (2020). In order to further clarify the biological significance of metabolites, metabolic pathway analyses were demonstrated, and the identified differential metabolites were visualized in a KEGG pathway plot.

### Statistical analysis

Fermentation profiles are reported as the mean and the standard error of the mean (SEM). The effect of ensiling time and inoculant on fermentation quality was analyzed by two-way analysis of variance using SPSS version 26.0 (SPSS INC., Chicago, IL, USA). *P* < 0.05 was considered statistically significant, and then the group means were further compared with Duncan's multiple range tests. The bioinformatics analyses of the bacterial community were mainly performed using the QIIME and R software (V 4.0.0). Projections to latent structure-discriminant analysis (PLS-DA) models were tested for metabolites. Variable importance in projection (VIP) ≥ 1.0 and *P* < 0.05 was used as a criterion for differential metabolite selection.

## Data Availability

All data generated or analyzed in the present study are included in this article.

## Abbreviations

AA: Acetic acid
ADF: Acid detergent fiber
BA: Butyric acid
CK: Control,
DM: Dry matter
FM: Fresh material
HE: *Lactobacillus buchneri* TSy1-3
HO: *Lactobacillus rhamnosus* BDy3-10
LA: Lactic acid
LAB: Lactic acid bacteria
M: Combination of HE and HO
NDF: Neutral detergent fiber
OTUs: Number of operational taxonomic units
PA: Propionic acid
PCoA: Principal coordinate analysis
PLS-DA: Partial least squares-discriminate analysis
WSC: Water-soluble carbohydrates
